# Spatial Transcriptomic Analysis of Focal and Normal Areas of Myocyte Disarray in Human Hypertrophic Cardiomyopathy

**DOI:** 10.3390/ijms241612625

**Published:** 2023-08-10

**Authors:** Jason Laird, Gayani Perera, Rebecca Batorsky, Hongjie Wang, Knarik Arkun, Michael T. Chin

**Affiliations:** 1 Research Technology, Tufts University, Medford, MA 02144, USA; jason.laird@tufts.edu; 2 Molecular Cardiology Research Institute, Tufts Medical Center, Boston, MA 02111, USA; gayani.perera@tuftsmedicine.org; 3 Data Intensive Studies Center, Tufts University, Medford, MA 02155, USA; rebecca.batorsky@tufts.edu (R.B.); hongjie.wang@tufts.edu (H.W.); 4 Department of Pathology, Tufts Medical Center, Boston, MA 02111, USA; karkun@tuftsmedicalcenter.org

**Keywords:** hypertrophic cardiomyopathy, spatial transcriptomics, single-nucleus RNA sequencing, gene expression, bioinformatics, cardiovascular disorder, genetic disorder

## Abstract

Hypertrophic Cardiomyopathy (HCM) is a common inherited disorder that can lead to heart failure and sudden cardiac death, characterized at the histological level by focal areas of myocyte disarray, hypertrophy and fibrosis, and only a few disease-targeted therapies exist. To identify the focal and spatially restricted alterations in the transcriptional pathways and reveal novel therapeutic targets, we performed a spatial transcriptomic analysis of the areas of focal myocyte disarray compared to areas of normal tissue using a commercially available platform (GeoMx, nanoString). We analyzed surgical myectomy tissue from four patients with HCM and the control interventricular septum tissue from two unused organ donor hearts that were free of cardiovascular disease. Histological sections were reviewed by an expert pathologist, and 72 focal areas with varying degrees of myocyte disarray (normal, mild, moderate, severe) were chosen for analysis. Areas of interest were interrogated with the Human Cancer Transcriptome Atlas designed to profile 1800 transcripts. Differential expression analysis revealed significant changes in gene expression between HCM and the control tissue, and functional enrichment analysis indicated that these genes were primarily involved in interferon production and mitochondrial energetics. Within the HCM tissue, differentially expressed genes between areas of normal and severe disarray were enriched for genes related to mitochondrial energetics and the extracellular matrix in severe disarray. An analysis of the gene expression of the ligand–receptor pair revealed that the HCM tissue exhibited downregulation of platelet-derived growth factor (PDGF), NOTCH, junctional adhesion molecule, and CD46 signaling while showing upregulation of fibronectin, CD99, cadherin, and amyloid precursor protein signaling. A deconvolution analysis utilizing the matched single nuclei RNA-sequencing (snRNA-seq) data to determine cell type composition in areas of interest revealed significant differences in fibroblast and vascular cell composition in areas of severe disarray when compared to normal areas in HCM samples. Cell composition in the normal areas of the control tissue was also divergent from the normal areas in HCM samples, which was consistent with the differential expression results. Overall, our data identify novel and potential disease-modifying targets for therapy in HCM.

## 1. Introduction

Hypertrophic Cardiomyopathy (HCM) is an inherited disorder affecting between 1 in 500 and 1 in 200 people (OMIM 192600, 115195, 115196, 115197 and others). The disease is characterized by unexplained left ventricular hypertrophy that is often asymmetric, involves the interventricular septum, and is associated with left ventricular outflow tract (LVOT) obstruction, fibrosis, microvascular occlusion, and sudden cardiac death. Histologically, it is characterized by focal areas of myocyte hypertrophy, myocyte disarray, fibrosis and medial hyperplasia. Anatomically, it is characterized by mitral valve abnormalities and left ventricular outflow tract obstruction. Physiologically, it is characterized by enhanced contractile function, reduced diastolic function and increased risk of sudden cardiac death [1]. Traditionally, HCM is considered a disease that ensues from sarcomere gene dysfunction, but in most patients, pathogenic sarcomere gene mutations cannot be identified. In those patients where pathogenic gene mutations are found, most are located in the sarcomere genes *MYBPC3* and *MYH7*. The genetic landscape of HCM is well-summarized [2]. The activation of signaling pathways that promote cardiac myocyte hypertrophy and fibrosis of the heart have been implicated in many studies [3], but additional mechanisms are likely contributing. Comprehensive studies to understand how sarcomere gene mutations can lead to phenotypes not related to sarcomere function or those seen in cells that do not express sarcomere genes are lacking in the field. Since sarcomere gene mutation-negative patients have similar phenotypes to sarcomere gene mutation-positive patients, it is likely that there are final common pathological pathways independent of sarcomere gene mutations that are involved, but these final common pathways are incompletely understood. Recent reports using single-nucleus RNA sequencing of human HCM tissue have identified potential alterations in cell-to-cell communication involving extracellular matrix proteins, integrin receptors and the activation of immune cells as potential contributors to the HCM phenotype [4,5,6]. These studies, however, did not determine how these alterations in single-cell transcription and intercellular communication are spatially organized in the context of known histopathological features of HCM. To identify changes in gene expression associated with focal areas of myocyte disarray, we performed a spatial transcriptomic analysis of genes expressed in these areas that included the identification of differentially expressed genes (DEGs), a gene ontology (GO) analysis to assign DEGs to molecular function, a Ligand-Receptor (L-R) gene expression analysis to infer cell–cell communication and a deconvolution analysis to determine the cell-type composition in these areas. Here, we report that areas of focal myocyte disarray show an altered expression of genes involved in interferon production, mitochondrial energetics and the extracellular matrix that may also reflect changes in cellular composition. Furthermore, these areas also show dysregulation of PDGF and cadherin signaling that may be relevant to the pathogenesis of HCM.

## 2. Results

### 2.1. Identification of Focal Areas of Myocyte Disarray and Designation of Regions of Interest

**Figure 1:**
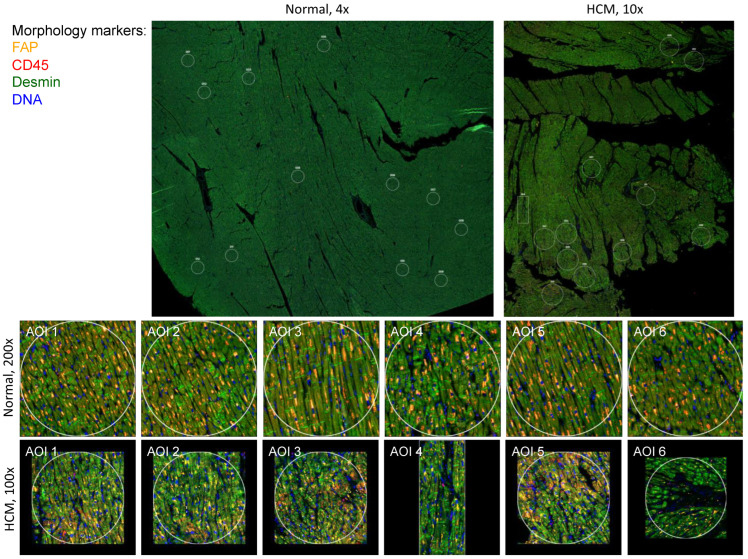
Human heart tissue sections used for spatial transcriptomic analysis. Normal and HCM histological sections from the interventricular septum were immunostained for FAP, CD45, Desmin and nuclear DNA, as described in Methods. Representative samples 2799 (Normal) and 2879 (HCM) are shown at lower magnification, along with designated Areas of Interest (AOIs) that were chosen based on the degree of myocyte disarray. The first 6 AOIs for each sample are shown at higher magnification. Myocyte disarray is apparent at higher magnification in the HCM samples.

HCM and control patient sections were stained with morphology markers desmin, fibroblast activator protein, CD45 and nuclear DNA using Syto83, as described in Methods. Representative images from HCM sample 2799 and control sample 2879 are shown in Figure 1. Representative Regions of Interest (ROIs), also called Areas of Interest (AOIs), are indicated and shown at a higher magnification.

### 2.2. Identification of Differentially Expressed Genes and Associated Pathways in Areas of Disarray

**Figure 2:**
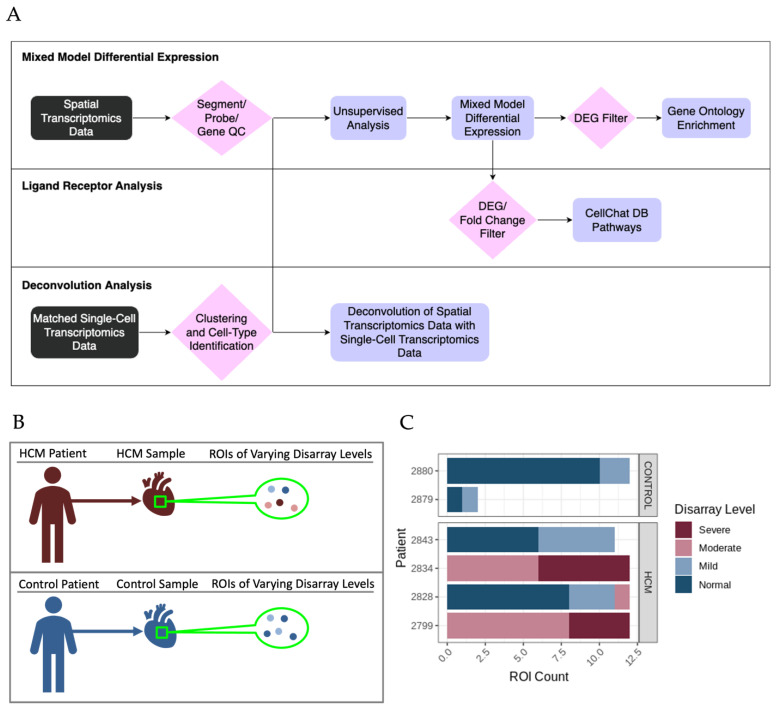
Study design and overall analysis pipeline for spatial transcriptomic data from HCM patient tissue. (**A**) Overall analysis pipeline for spatial transcriptomic data. (**B**) Overview of the hierarchy between patients, samples and ROIs. (**C**) Distribution of ROIs that passed quality control between patients and colored by disarray level.

**Figure 3:**
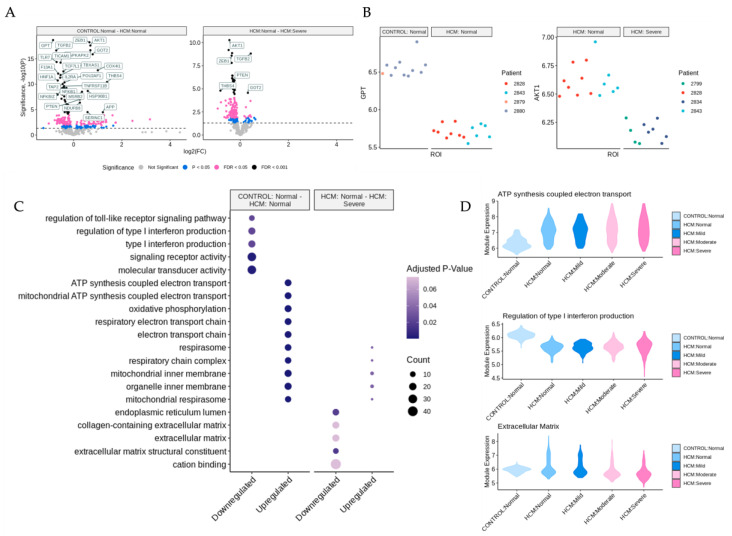
Identification of differentially expressed genes and associated biological processes in areas of myocyte disarray. (**A**) Volcano plots comparing areas of normal myocyte disarray between control and HCM patients and the progression of increasing myocyte disarray within the HCM phenotype. In each panel, gene expression fold changes are shown in the condition listed second in the plot title relative to the condition listed first. Only differentially expressed genes that had an FDR-adjusted *p*-value below 0.05 were considered differentially expressed. (**B**) Intra-patient variability in gene expression of the top differentially expressed genes for the comparison shown in (**A**). (**C**) Gene ontology enrichment dot plot of significant terms per comparison. (**D**) Module expression of significant annotations broken down by HCM status and myocyte disarray.

The bioinformatic analysis pipeline for the identification of differentially expressed genes (DEGs) between sets of ROIs and associated functional enrichment is shown schematically in Figure 2A. DEGs were determined in pairwise comparisons between sets of ROIs classified by the degree of myocyte disarray and patient HCM status (Figure 3A, Supplemental Table S1). Given the low number of ROIs that passed quality control, areas of mild disarray from the control patients were excluded from further analysis. We focused on two comparisons: normal disarray ROI between HCM and control patients, as well as between normal and severe disarray ROIs within HCM patients. The largest number of DEGs was obtained in the comparison of normal disarray ROIs between HCM and control patients, suggesting that the HCM macroanatomic phenotype itself is associated with large changes in gene expression, while fewer DEGs were found in the comparison between normal and severe disarray levels in HCM patients (Figure 3A and Figure S1A). The visualization of intra-individual variation in gene expression in the top differentially expressed genes confirmed that gene expression differences were driven by phenotype or disarray level rather than by individual patient identity (Figure 3B). Gene Ontology enrichment analysis revealed an upregulation in genes related to mitochondrial energetics and a downregulation of genes involved in interferon production in HCM ROIs of varying levels of disarray compared to normal control ROIs (Figure 3C and Figure S1B). Within HCM patients, severe disarray ROIs showed a weaker upregulation in genes associated with mitochondrial energetics and downregulation in genes that were constituents of the extracellular matrix when compared with normal disarray ROIs (Figure 3C). Interestingly, there was a large overlap between DEGs obtained in the comparison of the control normal and HCM normal ROIs and other comparisons between the control normal and HCM with more severe disarray (Supplemental Figure S1A). This overlap was even more pronounced in the GO categories obtained by performing GO enrichment of the DEGs obtained from these comparisons (Supplemental Figure S1B). This suggests that the HCM macroanatomic phenotype itself is associated with strong gene expression changes independent of the microanatomic severity of disarray.

### 2.3. Identification of Potentially Altered Cell–Cell Interactions in Areas of Myocyte Disarray

**Figure 4:**
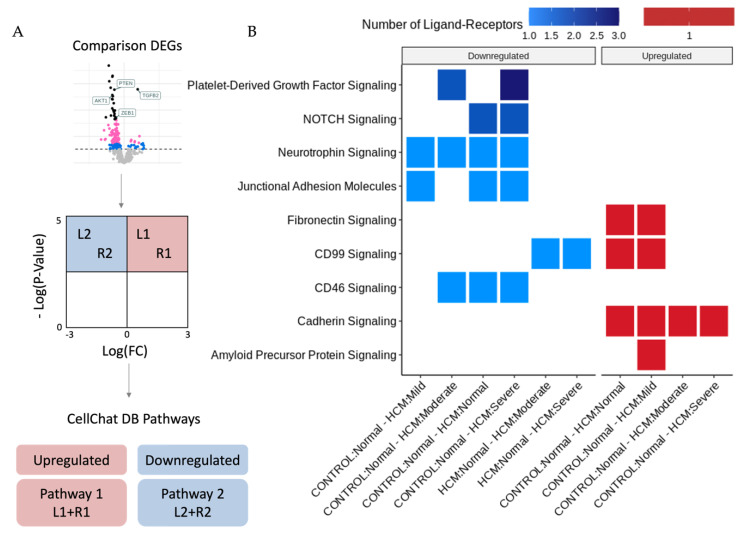
Identification of differentially expressed ligand receptors in areas of myocyte disarray. (**A**) Differentially expressed genes between myocyte disarray levels were filtered by an FDR-adjusted *p*-value of 0.05 and split into upregulated and downregulated groups. These gene sets were then compared to the CellChat database to identify Ligand–Receptor pairs and their associated pathway. (**B**) Heatmap indicating which pathways were affected in different disarray level comparisons and colored by the number of ligand receptors present in the pathway.

The bioinformatic analysis pipeline for the identification of Ligand–Receptor (L–R) pairs that are differentially expressed in areas of myocyte disarray is shown schematically in Figure 2A and further explained in Figure 4A. Differentially expressed genes were determined in pairwise comparisons between ROIs classified by the degree of myocyte disarray and overall patient HCM status, as described above. The gene sets from each comparison were separated by whether they were down or upregulated. The gene sets were then compared to the CellChat Interaction Database [7] to identify Ligand–Receptor pairs that were both present in either the up or downregulated gene sets and which pathway the Ligand–Receptor pair was associated with (Supplemental Table S2). Here, we note that, like the differential expression results, significant Ligand–Receptor pairs are observed when comparing ROIs obtained from HCM patients with varying levels of disarray to ROIs obtained from control patients. Although the GO enrichment analysis of the DEGs between control normal ROIs and HCM ROIs with varying levels of disarray did not uncover differences in GO terms, there were differences in the CellChat pathways associated with significant ligand–receptor DEG pairs. In HCM patients, compared to the control patients, normal disarray areas show a downregulation of CD46, junctional adhesion molecule, neurotrophin, and NOTCH signaling, while cadherin, CD99 and fibronectin signaling are upregulated. Areas of mild disarray in HCM patients are downregulated for junctional adhesion molecule and neurotrophin signaling and upregulated for cadherin, CD99, fibronectin and amyloid precursor protein signaling compared to normal disarray areas in control patients. Moderate disarray areas in HCM patients are downregulated for CD46, neurotrophin, and platelet-derived growth factor signaling and upregulated for cadherin signaling compared to normal disarray areas in control patients. Severe disarray areas in HCM patients are downregulated for CD46, junctional adhesion molecule, neurotrophin, NOTCH, and platelet-derived growth factor signaling and upregulated for cadherin signaling compared to normal disarray areas in control patients. Interestingly, CD99 signaling is upregulated in the HCM normal/mild ROIs compared to the control normal ROIs but downregulated in the HCM moderate/severe ROIs compared to the HCM normal ROIs. Also, JUN kinase signaling is upregulated in areas of severe disarray compared to areas of moderate disarray. The greatest number of Ligand–Receptor pairs driving these pathways is observed in platelet-derived growth factor signaling, followed by NOTCH signaling. All other pathways have one Ligand–Receptor pair per disarray level comparison.

### 2.4. Determination of Cell-Type Composition in Areas of Myocyte Disarray

**Figure 5:**
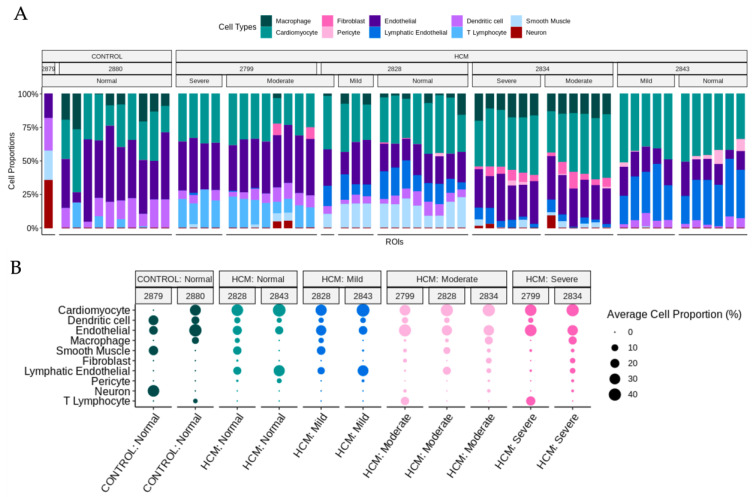
Cell-type composition of areas of focal myocyte disarray determined by deconvolution of SnRNA-seq Data. (**A**) Deconvolution of ROIs using per-patient averaged matched SnRNA-seq data and broken down by HCM status, patients and disarray level. (**B**) Average cell proportions of ROIs in different HCM status, disarray levels and patients.

Previously published snRNA-seq datasets from the patients in this study were reanalyzed to determine cell-type compositions [4,5,6,8]. UMAP plots and a dot plot showing cell clusters and cell identity assignments separated by disease label are shown in Supplemental Figure S2. To determine whether the cell type composition differs in areas of myocyte disarray compared to the normal and in HCM vs. control, we performed a deconvolution analysis (Methods) [9] using genes present in both the snRNA-seq data and the spatial data (Supplemental Table S3, Figure 5A). Note that areas of moderate and severe disarray were only present in ROIs obtained from HCM patients. To get a more granular picture of cell-type composition, the average cell proportion for each observed cell type was broken down by HCM status, disarray level and patient (Figure 5B). We note that the proportion of cardiomyocytes, dendritic cells and endothelial cells were relatively constant across patients, HCM status and disarray levels (Figure 5B). On the other hand, fibroblast proportions increased as the severity of disarray increased (Figure 5B). ROIs from HCM patients with normal and mild levels of disarray contained more lymphatic endothelial cells (Figure 5B). The proportions of macrophages, smooth muscle, neuronal cells and T-lymphocytes did not show consistent changes in proportions across patients within a HCM status/disarray level (Figure 5B). 

We also assessed whether cell-type diversity differed between the ROI from the control and HCM patients using the cell-type diversity statistic described by Karagiannis et al. [10]. Although there is a trend towards higher cell-type diversity in HCM patients compared to control patients, the results were not statistically significant at the *p* < 0.05 level when accounting for inter-patient variability via a linear mixed-effect model (Supplemental Figure S3A).

## 3. Discussion

Spatial transcriptomics can be used to map transcriptional patterns to specific anatomic locations [11,12,13] and can complement high-resolution, non-spatially resolved single-cell transcriptomic datasets by facilitating the mapping of diseased cell types to areas of pathological change through bioinformatic deconvolution methods [14,15]. Such approaches have been used to map areas of SARS-CoV-2 infection and lung injury [16,17] but, to the best of our knowledge, have not been used to study HCM tissues. Here, we report the spatial transcriptomic profiling of areas of focal myocyte disarray, lesions pathognomonic for HCM and thought to reflect the intrinsic pathophysiological processes inherent to diseased cells using a set of probes specific for the cancer cell transcriptome (nanoString, Seattle, WA, USA). We have found that focal areas of myocyte disarray specifically show changes in gene expression associated with interferon production, the extracellular matrix and mitochondrial function. These findings suggest alterations in pro-inflammatory and metabolic processes in areas of myocyte disarray, which may ensue from sarcomere dysfunction, often the primary disease-driving process in HCM. These findings are also consistent with previous studies noting altered interferon levels and inflammatory markers in HCM patients [18,19] and altered mitochondrial function in HCM patients [20,21]. Our study is unique, however, in that it specifically implicates these processes in areas of focal myocyte disarray, thus linking these processes to discrete histopathological defects. 

Analysis of ligand-receptor alterations provides a window into how intercellular communication may be altered in areas of focal myocyte disarray. The downregulation of CD46, junctional adhesion molecule, neurotrophin, NOTCH and PDGF signaling in areas of severe myocyte disarray, as shown in Figure 4, may reflect reduced complement inactivation (CD46) [22], reduced integrin-mediated leukocyte and platelet adhesion (junctional adhesion molecule) [23], reduced neuronal innervation (neurotrophin) [24], loss of cardioprotection (NOTCH) [25] and reduced smooth muscle and fibroblast proliferation (PDGF) [26] in these areas. Increased cadherin signaling implies increased cell adhesion [27], which may represent a response to increased mechanical force associated with HCM. Reduced inactivation of complement and reduced leukocyte and platelet adhesion imply alterations in the inflammatory response. Changes in neuronal homeostasis may imply altered autonomic innervation and the potentiation of arrhythmogenesis. Alterations in smooth muscle proliferation may also reflect altered vascularity in these areas, while alterations in fibroblast proliferation may reflect alterations in fibrosis. CD99 plays an important role in T-cell activation [28] and the suppression of extracellular matrix–integrinβ1 interactions relevant to cell adhesion [29]. Its upregulation in areas of mild disarray but downregulation in areas of moderate or severe disarray may reflect a role for T-cell immune function in early, mild lesions that is then dispensable in advanced lesions. Future studies targeting these specific pathways may lead to improved experimental and therapeutic outcomes.

Deconvolution analysis of snRNA-seq data in conjunction with spatial transcriptomic data facilitates the determination of specific cell-type composition within focal areas of myocyte disarray. As expected, the cell composition in areas of moderate or severe disarray and normal areas in HCM samples diverged, with areas of disarray showing a higher proportion of fibroblasts, consistent with altered fibrotic mechanisms in these areas. Normal areas in HCM tissue showed a higher proportion of lymphatic endothelial cells, suggesting that these areas are in a different physiological stage compared to areas of moderate or severe disarray. These findings raise an interesting question of whether the areas of focal myocyte disarray are anatomically distinct by virtue of differences in innervation, capillary density, and lymphatics, which may facilitate the differential and distinctive recruitment of immune-cell populations present in the different areas. In this model, the detection of focal myocyte disarray would, thus, likely be a local consequence of a more global disease process rather than an area of focal pathophysiology. Additional higher-resolution spatial transcriptomic studies with targeted deletion of specific cell populations, such as fibroblasts, would likely provide further insight. 

A recent study examined spatial transcriptomic patterns in Arrhythmogenic Cardiomyopathy (ACM) using Tomo-Seq and identified ZBTB11 upregulation in cardiomyocytes as a potential cause of autophagy and apoptosis in this disorder [30]. ACM and HCM differ significantly in terms of histopathologic features and genetic causes. We do not see the alterations in ZBTB11 expression, nor in genes relevant to autophagy or apoptosis in our study, but this is not surprising given the divergent features of these cardiomyopathies.

Limitations of our study include the small number of patient samples, the use of the Human Whole Cancer Transcriptome Atlas reagent set and the limited spatial resolution of the GeoMX technology. Spatial transcriptomic analysis, while powerful, is currently limited by expense and low throughput, and thus the sample number can be a limiting factor in determining DEGs and in determining cell composition. We mitigated the effect of a small sample size on determining DEGs by including a random patient effect in our mixed model, as described in Methods. The Cancer Transcriptome Atlas assesses approximately 1800 mRNA targets and is designed for the profiling of cancerous tumors and the tumor microenvironment and, thus, does not address the entire transcriptome and may not detect critical transcriptional pathways not included in the probe set. At the time this study was done, the Human Whole Transcriptome Atlas was not yet available. Future studies using this newer whole transcriptome atlas will likely be informative. Finally, the spatial resolution of the GeoMX technology is limited to ~100–200 cells and, thus, cannot truly provide single-cell resolution. The latest CosMX technology from nanoString can now provide single-cell resolution. Future studies using these newer technologies may provide even greater insights into the single-cell and spatial transcriptomic analysis of human HCM.

## 4. Materials and Methods

### 4.1. Patient Characteristics and SnRNA-Seq Datasets

Patients with HCM and the control patients without cardiovascular disease and their snRNA-seq datasets from the cardiac interventricular septum have been described previously [4,5,6,8]. De-identified samples from HCM patients 2799, 2834, 2828 and 2843 and the control patients 2879 and 2880 were used in this study. The snRNA-seq datasets are available in the Gene Expression Omnibus database under accession numbers GSE161921, GSE174691 and GSE181764. The HCM and control patients who provided tissues used in this study have been described extensively in prior publications [5,6]. Control patients 2879 and 2880 are described in [5], Table 2. The HCM patients 2799, 2828, 2834 and 2843 correspond to patients 1, 4, 6 and 9 in [6], Supplemental Table S1. Patient 2828 had a pathogenic *KRAS* mutation, and patient 2843 had a pathogenic *MYBPC3* mutation. 

### 4.2. Tissue Processing for Spatial Transcriptomics

Paraffin-embedded tissues were generated from each tissue sample and sectioned for spatial transcriptomic analysis using standard methods. The tissue sections were generated within 2 weeks of spatial analysis and were processed for spatial transcriptomics analysis according to the GeoMx Digital Spatial Profiling protocol [12], as provided by the manufacturer (nanoString, Seattle, WA, USA). Briefly, samples were stained for morphology using commercially available antibodies to desmin (Abcam, Cambridge, UK, cat. # ab185033) at 1:200 dilution, fibroblast activating protein (Abcam cat. # ab238148) at 1:50 dilution and CD45 (Cell Signaling Technology, Danvers, MA, USA, cat. # 13917BF) at 1:100 dilution. The nuclei were counterstained with Syto83 (ThermoFisher, Waltham, MA, USA). Tissue morphology was visualized for each tissue slide using the GeoMx Digital Spatial Profiler, and areas of focal myocyte disarray were designated as regions of interest (ROIs) by an expert pathologist. The ROIs were graded for the degree of myocyte disarray on a scale of severe, moderate, mild and normal. A total of 12 ROIs were selected from each tissue slide. The RNA within the ROIs was captured and profiled using the GeoMx Cancer Transcriptome Atlas (nanoString) to detect approximately 1800 RNA targets. Samples were processed in 2 batches, with the first batch consisting of 2 HCM and 2 normal samples and the second batch with 2 HCM samples. Serially sectioned slides stained with hematoxylin and eosin or trichrome were also done to aid in morphological assessment.

### 4.3. Identification and Analysis of Differentially Expressed Genes Associated with HCM Areas of Myocyte Disarray

Raw expression data from ROIs underwent quality control and Q3 normalization per the recommendations from the manufacturer (nanoString). Segment, probe, and gene quality control were performed using the R package, GeoMXTools v2.99.2. The expressed genes were filtered for inclusion in at least 1% of segments. Samples that passed quality control underwent unsupervised analysis to identify potential confounding factors. Linear mixed-effects models were used to test for differential gene expression between groups of ROIs with different levels of disarray and HCM status using a composite variable indicating HCM status and disarray level as a fixed effect and setting the patient identifier as the random effect. Genes with Benjamini–Hochberg adjusted *p*-values of less than 0.05 were considered significantly differently expressed between groups of ROIs. Gene Ontology analysis was performed using the R package, ClusterProfiler, using all three GO ontologies [31,32].

### 4.4. Ligand-Receptor Analysis to Delineate Potential Intercellular Communication Pathways That Promote Focal Myocyte Disarray

Differentially expressed genes were further analyzed for the presence of Ligand–Receptor pairs that were differentially expressed in the same way (e.g., either both upregulated or both downregulated, called differential combination analysis) [33] using known human ligand–receptor pairs present in the CellChat Interaction Database [7].

### 4.5. Deconvolution of Single-Nucleus RNA-Sequencing Data to Determine Cell Composition in Areas of Focal Myocyte Disarray

SnRNA-seq datasets from the six samples were integrated into a single Seurat object [34] using Harmony [35]. The optimal clustering resolution was determined using ChooseR [36]. Cell assignments were generated using the expression of canonical markers and methods described previously [4,5,6,8]. The snRNA-seq datasets were filtered to only include marker genes present in the GeoMx ROI data and log2 transformed. The GeoMx ROI data were Q3 normalized and log2 transformed before undergoing deconvolution. Spatial Deconvolution Analysis was used to determine the cellular composition of areas of focal myocyte disarray using SpatialDecon [9]. Deconvolution was performed on a per- patient basis, where each patient’s GeoMx ROI data were deconvoluted using the patient’s matching snRNA-seq data as a reference. The cell-type diversity statistic, described by Karagiannis et al. [10], was used to assess the cell-type diversity of ROIs of varying levels of disarray and HCM status. A linear mixed-effects model was used to test for differences in the cell-type diversity statistic value between ROIs of varying levels of disarray and HCM status using a composite variable indicating the HCM status and disarray level as a fixed effect and setting the patient identifier as the random effect.

## 5. Conclusions

Here, we report the first spatial transcriptomic analysis of human HCM samples, focusing on areas of focal myocyte disarray. These areas of focal myocyte disarray show distinctive changes in gene expression related to interferon production, mitochondrial metabolism and the extracellular matrix. Analysis of intercellular communication in these areas reveals significant changes in cell adhesion, PDGF, NOTCH and cadherin signaling. An analysis of the cell content in these areas reveals characteristic differences in lymphatic endothelial cells and fibroblasts. The characterization of the complex interplay between cells within HCM lesions will likely lead to the development of novel, targeted therapeutics, perhaps those that target interferon signaling or mitochondrial metabolism, to improve outcomes in HCM patients.

## Data Availability

Spatial transcriptomic data is available upon reasonable request.

## Supplementary Materials

The following supporting information can be downloaded at: https://www.mdpi.com/article/10.3390/ijms241612625/s1.

## Abbreviations

HCM	Hypertrophic Cardiomyopathy
LVOT	Left Ventricular Outflow Tract Obstruction
DEG	Differentially Expressed Gene
GO	Gene Ontology
L–R	Ligand–Receptor
ROI	Region of Interest
AOI	Area of Interest
FDR	False Discovery Rate
snRNA-seq	Single-Nucleus RNA-sequencing
UMAP	Uniform Manifold Approximation and Projection
DCA	Differential Combination Analysis
