# Supplementary material for: Spatial Transcriptomic Analysis of Focal and Normal Areas of Myocyte Disarray in Human Hypertrophic Cardiomyopathy
Source: Int J Mol Sci. 2023 Aug 10;24(16):12625. doi: 10.3390/ijms241612625 (PMC10454036; doi:10.3390/ijms241612625)
Supplement: Supplementary file 1 [file ijms-24-12625-s001.zip › ijms-2513977-supplementary figures.pdf]

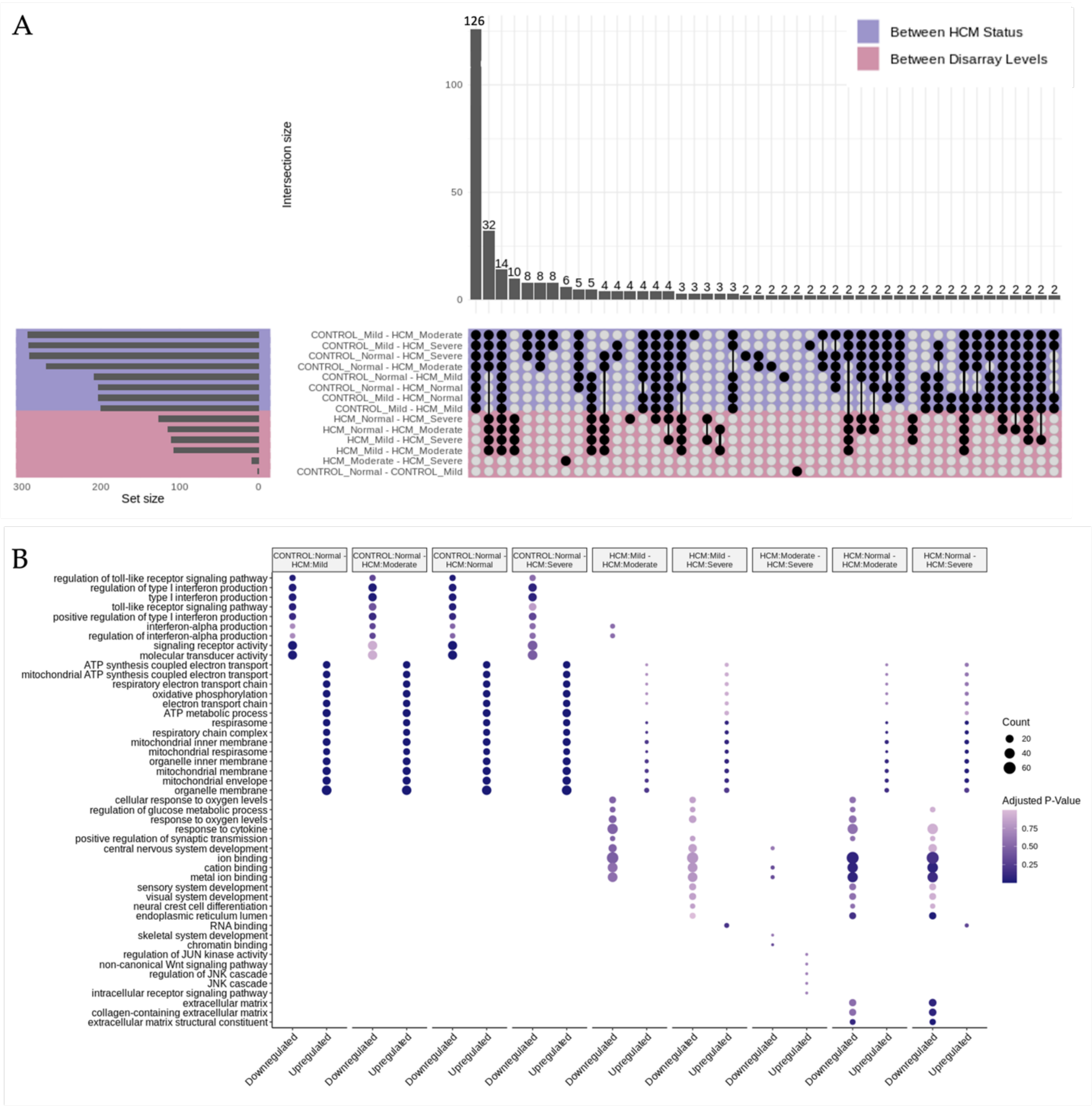

Supplemental Figure S1. Identification of Differentially Expressed Genes Between All Areas of Myocyte Disarray. **A.** UpSet plot revealing the differentially expressed gene overlap between all disarray comparisons. Comparisons between control patients and HCM patients are highlighted in purple and comparisons within HCM status are highlighted in pink. **B.** Gene ontology enrichment dot plot of top annotations per comparison for all disarray comparisons. Only differentially expressed genes that had an FDR adjusted p-value below 0.05 were enriched per disarray comparison. **C.** Top differentially expressed gene expression for each comparison of interest and broken down by patient, HCM status, and disarray level to assess any patient specific effects.

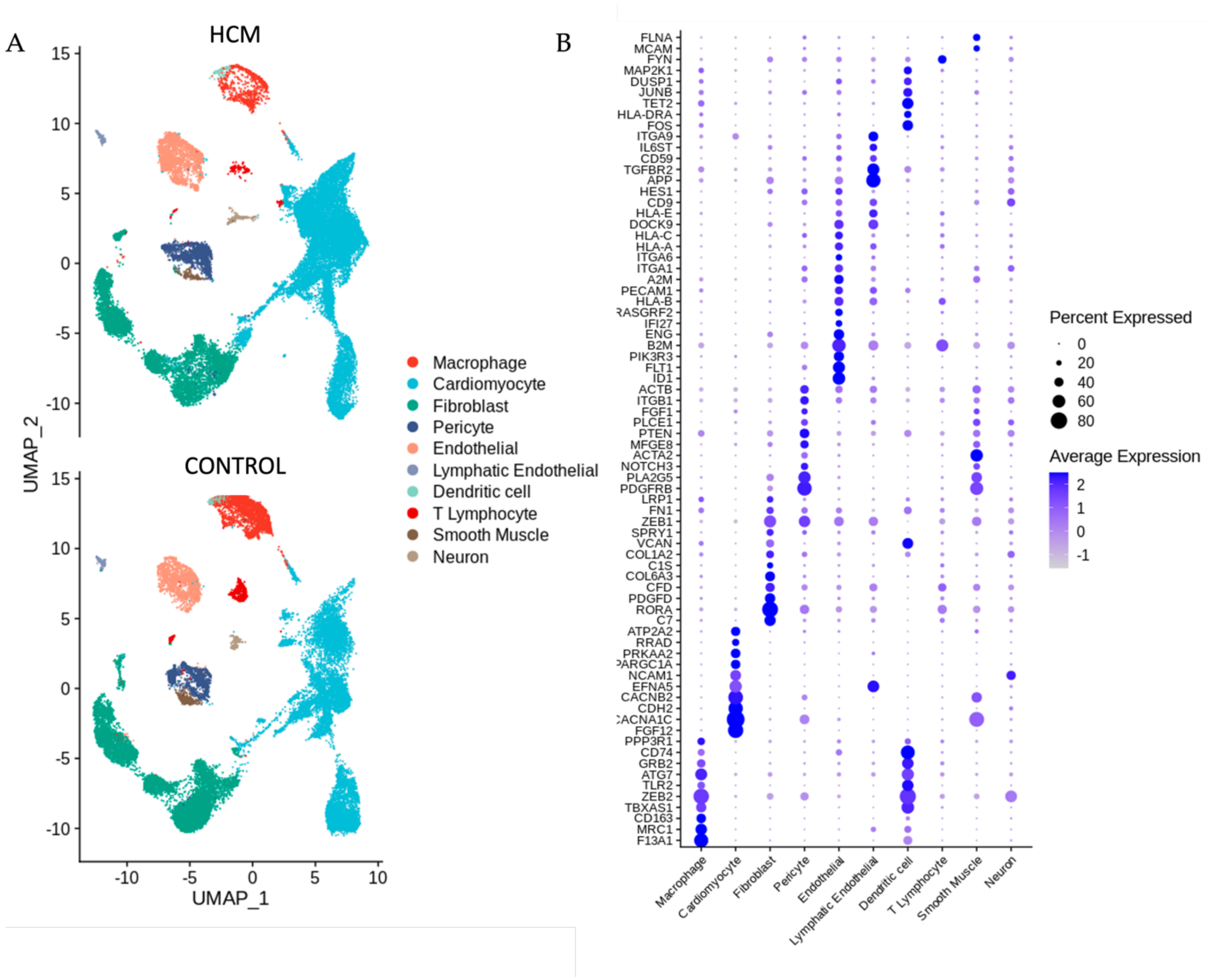

Supplemental Figure S2. Identification of Overlapping of Overlapping Cell Marker Genes Between GeoMx ROI data and Matched SnRNA-seq Data. A. UMAP plot showing cell clusters and cell identity assignments separated by disease label. **B.** Dot plot showing the expression of overlapping cell-type marker genes present in both the GeoMx ROI data and the matched SnRNA-seq data.

A

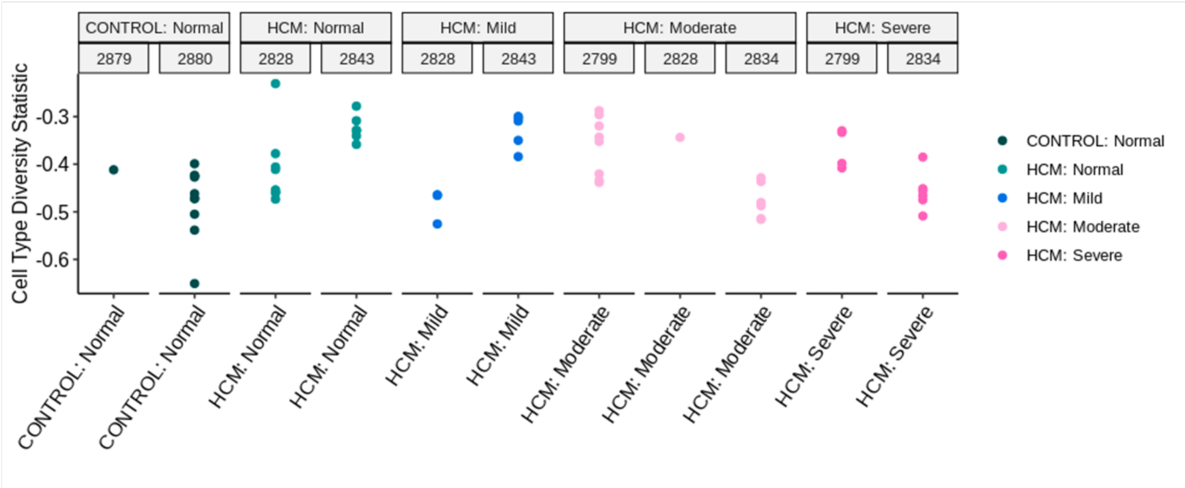

Supplemental Figure S3. Per Patient Cell type Composition of Areas of Focal Myocyte Disarray Determined by Deconvolution of SnRNA-seq Data. A. Cell diversity statistic summarizing the diversity of ROIs in different HCM status, disarray levels, and patients. Values closer to -1 indicate the sample is of one cell type and values closer to 0 indicate the sample is more varied in composition. There is a trend towards higher diversity in the HCM patients compared to Control patients, though the results were not statistically significant at the $p<0.05$ level when accounting for inter-patient variability via a linear mixed-effect model.
